# In Search of a Unifying Concept in Human Diseases

**DOI:** 10.3390/diseases9040068

**Published:** 2021-10-04

**Authors:** James Edward Trosko

**Affiliations:** Department of Pediatrics/Human Development, College of Human Medicine, Michigan State University, East Lansing, MI 48824, USA; trosko@msu.edu

**Keywords:** personalized medicine, artificial intelligence, precision oncology, multi-stage, multi-mechanism process of carcinogenesis, adult stem cells, gap junctional intercellular communication, turing algorithm, epigenetics, cancer drivers

## Abstract

Throughout the history of biological/medicine sciences, there has been opposing strategies to find solutions to complex human disease problems. Both empirical and deductive approaches have led to major insights and concepts that have led to practical preventive and therapeutic benefits for the human population. The classic definitions of “science” (to know) has been paired with the classic definition of technology (to do). One knew more as the technology developed, and that development was often based on science. In other words, one could do more if science could improve the technology. In turn, this made possible to know more science with improved technology. However, with the development of new technologies of today in biology and medicine, major advances have been made, such as the information from the Human Genome Project, genetic engineering techniques and the use of bioinformatic uses of sophisticated computer analyses. This has led to the renewed idea that Precision Medicine, while raising some serious ethical concerns, also raises the expectation of improved potential of risk predictions for prevention and treatment of various genetically and environmentally influenced human diseases. This new field Artificial Intelligence, as a major handmaiden to Precision Medicine, is significantly altering the fundamental means of biological discovery. However, can today’s fundamental premise of “Artificial Intelligence”, based on identifying DNA, as the primary nexus of human health and disease, provide the practical solutions to complex human diseases that involve the interaction of those genes with the broad spectrum of “environmental factors”? Will it be “precise” enough to provide practical solutions for prevention and treatments of diseases? In this “Commentary”, with the example of human carcinogenesis, it will be challenged that, without the integration of mechanistic and hypothesis-driven approaches with the “unbiased” empirical analyses of large numbers of data, the Artificial Intelligence approach with fall short.

## 1. Introduction: In Search of a Biological El Dorado

“Personalized medicine is the latest promise of a gene-centered biomedicine to provide custom-tailored to the specific needs of patients. Although surrounded by much hype, personalized medicine lacks the empirical and theoretical foundations necessary to render it a long-term perspective. In particular, the role of genetic data and the relationship between causal understanding, prediction, prevention and treatment of a disease need clarifying.” [1] “We resemble our progenitors because we derive from them our genetic endowment; but our genes do not determine traits by which we know a person. They only govern the responses that the person takes to the environmental [dietary] stimuli. Individuality progressively emerges from those responses [2].”

In an age where powerful technologies are available to biologists, as well as a shift in the approach to solve complex biological problems from a “biased, mechanistic hypothesis-driven approach” to an “unbiased” inductive examination of tons of data derived from these technologies, we are now incentivized to find a “***Rose in a dung heap***”. This is seen with the US NIH starting an initiative on “Precision Medicine” [3].

Even more disturbing to this author is that, in spite of early concepts that started the discipline of physiology by Claude Bernard and the discipline of artificial intelligence by Alan Turning [4], a very important fundamental biological process to help maintain homeostatic control of cell proliferation, cell differentiation, apoptosis, gene regulation and aging, namely, epigenetics, has been largely ignored by those championing the current precision medicine and artificial intelligence approach. The epigenetic mechanisms which involve the integration of extracellular, intracellular and cell–cell communication must be accounted for.

When Claude Bernard gave an explanation to whole animal responses by suggesting individual cells could send a molecular signal that could traverse the body to trigger a biochemical response to that molecular signal (e.g., hormones, growth factors, cytokines, chemokines, etc., or extracellular communication) could elicit in the specific cells receiving that signal, intracellular communication pathways that could determine the physiological fate of the receiving cell. What Dr. Claude Bernard did not know at the time was that there existed another system of communication, namely, cell–cell communication or intercellular communication, discovered by both electrophysiological techniques [5] and freeze fracture electron microscopy [6].

So, it was with Alan Turning amazing computer algorithm to explain biological patterning, such as spots on leopards or stripes on zebras via his “diffusion-reaction” model. He apparently had no access to the later discovery of gap junctional intercellular communication (GJIC). In addition, he had no biological training, nor was he even aware of the physiological model of cells talking to each other. His algorithm basically accounted for the two early models of cell- cell communication, namely, extra- and intracellular communication. This model is still being used to make fairly accurate predictions of biological patterning, but it is obviously not perfect.

Clearly, I am not saying that Artificial Intelligence or that the Turing model cannot be used to find “patterns” by using various algorithms on large data sets in an “unbiased” manner. The point I am trying to make is that these new patterns that are found give absolutely no insight to the underlying mechanism of the medical problem being studied. It is absolutely critical that mechanisms are understood, since, without this understanding, either no real practical outcome or even great human harm would be the result. In the case of human cancers, within the multi-stage, multi-mechanism process, it is experimentally clear that the initiation of that single normal stem cell is the result of a mutagenic event, either by an “error of DNA repair” or by an “error of DNA replication”. Knowing which type of mechanism causes that initiating event would be critical for basing any policy of intervention. In addition, the mechanism involved in the promotion process is clearly an epigenetic, non-mutagenic process. Knowing the detailed molecular, biochemical and cellular mechanisms is critical because these promoting properties can have quite diverse consequences, such as under certain conditions they can have antioxidant properties but under different conditions they can have pro-oxidant properties. The same chemical could be either a promoter or anti-promoter. The “progression” process is far more complex, involving either or both mutagenic or epigenetic.

I have tried to restrict my analysis of “Artificial Intelligence” or Turing-Type models to the understanding of human carcinogenesis. Since all human diseases involve the disruption of the integrated extra-, intra- and gap junctional intercellular communication processes, to my knowledge, to date, these models have yet to predict, in a “Precision Medicine” or “Personized Medicine” manner, any known disease. I make this judgement based on the biology of all pathologies of metazoans involve the integration of all three forms of cell communication. This is true whether it is a birth defect, carcinogenesis, cardiovascular pathologies, immune disorders, reproductive- and neurological pathologies.

Since I mentioned the immune process, which is highlighted in many current approaches to cancer therapies, I must bring a known fact of the relationship of the “initiated” cell and the surrounding normal cells that keep the “initiated” in a suppressed condition. It is obvious from classic initiation/promotion experimental studies that the immune system does not detect these single initiated cells. If it did, no one of us would ever get cancer. That is because the initiated single cells are being suppressed by the communicating surrounding normal cells. This phenomenon changes the “initiated” cell so that its phenotype appears “normal” to our immune system. This is a known fact, since an initiate cell can exist in this state until an epigenetic agent blocks that cell–cell suppressing effect, allowing that initiated cell to proliferate and not die by apoptosis. Most humans have many initiated cells in many of our organs, but they can reach death before any tumor is detected. Our skin has many sun-induced initiated cells. Skin cancer is the most predominate cancers. Yet, there are humans who live long lives without getting skin cancers. Yet, a non-artificial intelligent approach to study human carcinogenesis, based on hypothesis- testing of a “biased” potential series of mechanisms, has strong validated experimental support for initiated cells to remain in a quiescent state.

One last issue also has to be built in any machine learning or Artificial Intelligent model. Classic pathologists have long noted that cancers seem to fall into two categories, very “embryonic-like” or the differentiated types. Most of the solid tumors have two types of cancers, carcinomas and sarcomas. Some examples include basal or squamous carcinomas of the skin; small cell lung cancers and non-small cell lung cancers; partially differentiated polyp-type colon cancers and very “embryonic-like “flat type” colon cancers. This might suggest that these types of cancers have two different “initiated” cells, one that exist in the stem cell state at the type of initiation, while the other might have just start to differentiate at the initiation state. Within each type there can be phenotypic classifications, such in the skin, where Merkel cell carcinomas, Kaposi sarcoma, cutaneous skin lymphoma, skin adnexal tumors can be found, which suggests that the micro-environment of the “initiated” cell can influence the phenotype of the resulting cancer. All of these biological facts have come out of a biased hypothesis testing of the potential mechanism of each state of the carcinogenetic process. These biological facts have to be taken into account in any Artificial Intelligent model.

Current attempts by computer modelers are being made to improve the Turing algorithm, with only a few recognizing that this third form of cell–cell communication must be integrated into the Turning algorithm [7]. Recent attempts to understand how various genes detected from large data basis via artificial Intelligence and machine learning have been suggested [8,9,10]. The recent paper by Furman et al. [10] did suggest with their machine learning strategy that “mechanisms” could come out of their approach. While as a biologist, I am not able to understand the computer logic and mathematics that their model entails, the term “mechanisms” used in their paper does not compute with my biological understanding of the mechanisms of carcinogenesis. More communication between the scholars of Artificial Intelligence with those who have access to decades of mechanistic hypothesis testing, using both experiment and epidemiological studies, is called for.

## 2. A Multi-Cellular Organism Is a 3-D Organism

From the existence of the single cell organism during the early evolution of life on earth in a low oxygen environment to the eventual appearance of oxygen-producing microorganisms to the early multicellular organism, a large number of genes and physical/physiological structures and functions had to appear when the environment became rich with potentially toxic oxygen to signal cell organisms but important regulatory functions in multicellular organisms, such as the metazoans. Genes had to appear via evolution to protect genomic DNA from oxygen-derived metabolites that could interact with all biological macromolecules (drug metabolizing enzymes, antioxidants, DNA repair mechanisms, etc.); genes coding for nuclear membranes, specific regulation of patterns or sets of gene regulation; formation of a new cell type; i.e., stem cells; genes that code for intercellular direct communication or “gap junctions”; genes that control senescence; and genes that code for extracellular matrices and extracellular adherence molecules [11].

To be sure, single cells can communicate with other single cells in an aqueous environment. Their survival as an individual cell or as a species did not necessitate their direct contact. Their means of survival in an ever-changing environment only depended on symmetrical cell division, where errors of DNA repair or DNA replication might produce a new mutation that gave that one cell in this population to survive.

When the first multicellular metazoan appeared, that first cell would divide symmetrically until it reaches a small hollow ball shaped entity. At that moment, each cell had equal access to the outside world and to the inside world. However, at the next cell division, these new cells, by being forced inward from the surface, whereby, they no longer had equal access to the outside world. Their micro-environment had changed. This, then, forced new gene expression for its survival. This induced “differentiation”. In turn, this new series of cell division also had to adjust by being in its new micro-environment…and so on [12]. At this point, in this newly defined multicellular organism, a new mini microenvironment had to be created to maintain “stemness”. This new microenvironment for this new cell type was the ***Stem Cell Niche*** [13]. It had to protect these new stem cells from oxygen which is a powerful inducer of differentiation or apoptosis. It tries to maintain a low oxygen level (~2–3%) [14]. For the metazoan, these stem cells are needed for expanded tissue growth; wound healing; replacement of death of cell, etc.

The key to understanding this complex homeostatic control of symmetric and asymmetric cell division of stem cells and their live-span limited progenitor cells seems to reside in a few critical genes. Oct4 is the so-called “Queen Bee” gene to maintain stemness. It is a redox sensitive gene [15]. In other words, a stem cell that is exposed to high levels of oxygen will switch from symmetric to asymmetric cell division, turn off the Oct4 gene, while at the same time, turn on the connexin gene(s), which is (are) required for differentiation [16].

## 3. Stem Cells, Gap Junctions Intercellular Communication (GJIC) in Normal and “Cancer” Cells

One of the early observations regarding GJIC was that “normal” cells had growth control or “contact inhibition”, and they could differentiation, apoptosis and senesce. On the other hand, cancer cells lost growth control or “contact-inhibition”, could not terminally differentiate or apoptosis normally, and had gained “immortality” and lost the ability to senesce. One key factor to help explain the difference between the “normal cell” and the cancer cell was either the presence or absence of GJIC [17].

At the time of Loewenstein’s hypothesis that cancer cells lacked functional gap junctions, he was not aware of the concept or biological characteristics of stem cells. His concept of “normal” cells did not include “normal stem cells”, let alone “normal adult organ-specific stem cells”. Only after the isolation of human embryonic stem cells [18,19], and later the induced pluripotent stem cells [20], did the genes of Oct4 become a critical biomarker for these stem cells. In addition, the demonstration that Oct4 was a redox sensitive gene started to suggest the evolutionary purpose of this gene during the transition from single cell organisms to the metazoan multicellular organism, including its role in regulating the switch from symmetrical to asymmetrical cell division. Yet, even later, with the isolation of human adult organ-specific stem cells, it was possible to see if they also expressed either Oct4 or connexin genes [21]. What was found, in spite of some contradictory claims to the contrary [22,23,24], these normal human adults, such as human adult breast stem cells [25], organ-specific stem cells did express Oct4, whereas their differentiated daughters shut down Oct4 but started to express members of the connexin gene family and have function GJIC.

Along with these observations, early in the study of gap junctions and cancer, a new insight was provided from the field of the mechanisms of carcinogenesis, namely the concept that carcinogenesis consisted of three vary distinct phases, the “initiation” step that involved the irreversible conversion of a normal cell to an “immortalized” but not tumorigenic cells; this was then followed by the “promotion” step, that involved the clonal expansion of that single “initiated” cell by agents that were not mutagenic but acted by “epigenetic” mechanisms. Finally, this clonally expanded “initiated” population has another series of either mutagenic or epigenetic processes or both, the “Progression” step [26,27,28], to acquire the so-call “Hallmarks of cancer” [29,30]. This “initiated stem cell” was believed to be caused by either a mutation via an “error of DNA repair”, as in the Xeroderma Pigmentosum case [31,32,33,34,35], or by an “error of DNA Replication”, as in the Blooms syndrome case [36].

**Figure 1:**
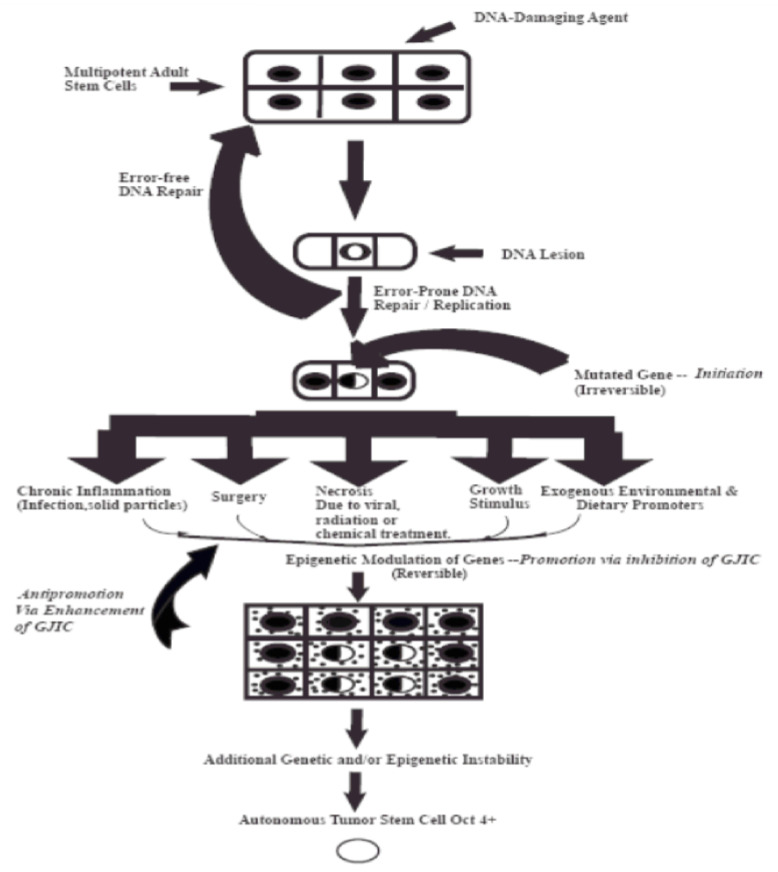
This diagram depicts the three steps of the multi-stage, multi-mechanism process of carcinogenes. The “initiation” step occurs in a single of a specific adult stem cell, characterized by expression the Oct4 gene) by either “an error of DNA repair” of DNA lesions induced by some DNA damaging agent, such as UV light or by an “error in DNA replication”. However, mutations in a few genes that influence the decision of the stem cell to divide either by symmetrical or asymmetriccal cell division do not confer a “driver” property. These initiated cells are suppressed by surrounding normal cells. It is the properties of non-mutagenic and “epigenetic” agents, such as growth factors, hormones, and cytokines, or conditions, cell rermoval or cytotoxic cell death, to infer with this suppressing effect that allows the clonal expansion of this initiated cell. Finally, after many cell divisions of this initiated clone, numerous other mutagenic and epigenetic changes occur in one of those initiated cells to acquire all the properties needed for invasion and metastatic property to become a “cancer stem cell” (the “Promotion” process).

In order to provide a visualization of this “initiation”/“promotion”/“progression” concept, which is based on validated experiment experiments, these three steps of carcinogenesis consist of very distinct mechanisms during the evolution of a normal cell (an adult organ-specific adult stem cell that exists in all organs) to become an invasive metastatic cancer stem cell. Figure 1 illustrates the fact that the “initiation” step of a normal adult stem cell is the result of some sort of an irreversible mutagenic mechanism. Important to note that all stem cells have the ability to divide either symmetrically to produce both daughters to maintain “stemness”, or to divide asymmetrically to produce one daughter that will go onto terminally differentiate, while the other daughter must maintain “stemness”. In the initial and predominate assumption of “precision oncology”, the “driver mutation” is thought occur at this stem. However, the initiation stem actually blocks symmetrical cell division and allows the “initiated cells” not to terminally differentiation. Yet, these mutations, probably only a very few, do not stimulate the proliferation of the initiated cells. That depends on external stimulation by non-mutagenic or “epigenetic” agents to release these “initiated cells” from the existing suppression of cell–cell communication of normal cells on these initiated cells. In other words, the “driver” component of this multi-stage, multi-mechanisms process of carcinogenesis is the “promotion” or epigenetic step.

Promotion mechanisms were shown to be triggered by agents that could reversibly inhibit gap junctional intercellular communication, such as growth factors, hormones, cytokines and chemokines. Other epigenetic chemicals inhibited pollutants (TCCD, DDT, PBB’s, PCB’s, TPA), etc.) [37]. These epigenetic tumor promoters had to exceed a threshold level, must be exposed to them at regular & extended or chronic times; and, also, be present in the absence of ant-promoters or anti-oxidants [38]. These epigenetic agents could be species-specific, gender-specific, developmental stage-specific and organ-specific [39]. The progression stage seems to be depended on either or both mutagenic or epigenetic agents to complete the steps needed to convert that “normal” cell to a “cancer cell”.

One other factor must be kept in mind. The “initiation” step is inevitable. It can occur at any time in any organ-specific cell. The risk to the initiation stem can be prevented to some extent, but it can never be completely eliminated. Every time a normal cell proliferates, an “error of DNA replication” could occur. On the other hand, the promotion step is the rate limiting step. It must exceed threshold levels, be present for regular and long periods of time, and in the absence of anti-promoters. Other agents that can modulate gap junction gene expression or function has been shown to be by several oncogenes (SRC, RAS, RAF, NEU) or by several oncogenic viruses, such as hepatitis and human papilloma virus). Genetic engineering of these genes into normal cells can convert these cells to become “immortal”, but not tumorigenic in one step. On the other hand, transforming non-communicating cancer cells with tumor suppressor genes or normal gap junction genes, results it the restoration of contact inhibition and the loss of the tumorigenic phenotype [40,41].

## 4. The Demonstration of the Role of Stem Cells in the Formation of “Cancer Stem Cells”

In the quest to understand the cancer process by either “artificial Intelligence” approach or the mechanistic hypothesis approach, it is fair to point out that, by using any algorithm to find patterns in mountains of data generated by powerful technologies, one will indeed find patterns. The question is “What will those patterns signify?” One major criticism, especially trying to understand carcinogenesis for the purpose of either prevention or treatment in the context of “Precision Medicine”, is that, unless those algorithms are based on some solid biological understanding, those patterns with offer little benefit. Case in point. Today, we already know that carcinogenesis consists of three distinct steps, each encompassing distinct molecular mechanisms, e.g., mutation; cell death, and epigenetic alteration of gene expression, transcription and translation. In addition, we also know that there are individual genetic backgrounds, gender, development stage, nutrient/dietary components; length of time exposures, environmental factors, medication, time of day exposure factor; life style behavioral and other psychological, social and cultural components that can influence the carcinogenic process. It seems very clear, that, unless any such AI algorithm to be used to answer the question as to what is the mechanism causing cancer, it must involve the incorporation of the multi-stage, multi-mechanisms process of carcinogenesis, as well as understanding the role of adult organ specific stem cells and the different mechanisms of mutagenesis or “initiation” and the properties and mechanisms of cell–cell communication.

Equally important is the question: “*What is the target normal cell such that, when initiated, ultimately gives rise to the cancer?*” That raises the issue of the two major opposing hypotheses of the origin of cancer, more specially, the “cancer stem cell”, namely, The “*Stem cell hypothesis*” [42,43,44] and the “*De-differentiation*” *or* “*Re-programming*” hypothesis [45]. These have been the two major hypotheses that need to be resolved. To be fair, it has not been universally accepted which of the two hypotheses is the correct one. However, without a comprehensive review of the experimental data to test these two hypotheses and the weight of the evidence on its side, for this “Commentary”, it will be assumed that the stem cell hypothesis seems to explain the origin of the cancer stem cell best.

Although intellectual arguments to support the Stem Cell hypothesis existed before the actual isolation and identification of human stem cells [18,19], several more recent experiments seem to support the Stem Cell hypothesis [46,47,48,49,50,51,52,53,54,55,56,57,58,59,60,61,62,63,64,65,66]. However, the recent demonstration that normal human adult breast stem cells, when treated with oncogenic virus and subsequent exposures to X-rays and genetically engineered one of the immortalized but non-tumorigenic derivative cells ultimately gave rise to a highly tumorigenic human breast “cancer stem cell” (See Figure 1 in [12]).

Indirect supporting evidence of some of the characteristics of the “cancer stem cell” came from the many observations of cells isolated from either real human tumors or cell lines derived from those tumors. The technique of “side population” cells was use on these tumor derived cells, in which the fluorescent, Hoechst 33342 stain, was exposed to all cells, then separated by flow cytometry based on whether the cells contained the dye or not [67]. It turned out that the small fraction of the tumor-derived cells or cells derived from the tumor, which contained no dye, these cells could sustain the long-term growth of the tumor (The operational definition of a “*cancer stem cells*”). Those florescent-containing cell were unable to sustain the long-term growth of the tumor. These were the “*cancer non-stem cells*” of the tumor or tumor cell line.

Another, source of support comes from the use of antibodies to the Oct4 transcript factor protein. If those normal adult organ-specific stem cells had Oct4 expressed and maintained its expression during the initiation, promotion, progression process, including in the “cancer stem cells”, this provided evidence that the Oct4 gene was not turned on or “re-programmed” from a differentiated adult somatic cell. This argument has been made related to the Noble Prize winner Dr. S. Yamanaka’s discovery of “Induced pluri-potent” stem cells (“iPS”) [68]. The argument goes as follows. If his interpretation of the isolation of his rare “iPS” cells after transfection of his embryonic genes (c-Myc, Klf4, Oct4, and Sox), was correct, then during the carcinogenic process in vivo must involve first, the “re-programming” of a single somatic differentiated or mortal cell to a “induced pluri-potent” stem cell, which operationally, has the potential, when in vivo, to be transformed to form the three germ layers or teratoma. If that is correct, then, “Why *are the majority of human tumors* in vivo *sarcomas and carcinomas, are not teratomas?”*

The argument against his interpretation is that he & his team never took into account that any primary cell line has a few adult organ-specific stem cells in its population [69]. These rare stem cells in that population are naturally “immortal” until they are induced to differentiate. So, when these 4 embryonic genes are added to all the cells in this primary cell population, containing many differentiated somatic “mortal” somatic cells and a few “immortal” adult stem cells, only the few stem cells survive with their endogenous Oct4 gene expressed, plus the exogenous embryonic genes, to be characterized as “iPS” cells. In reality, these “iPS” cells are not the “re-programmed” somatic mortal cells of that population, but the few naturally “immortal” stem cells. Even detailed examination of these so-called “iPS” cells that showed the genes of the differentiated tissue were still expressed in these “iPS” cells [70,71]. It was interpreted as showing that “re-programming” is never “complete”. Alternatively, it only demonstrates these so-called” “iPS” cells were originated from the adult stem cells of that tissue and that normal adult stem cells, which endogenously expresses the Oct4 gene, gave rise to the “cancer stem cells”, not by “re-programming”, but by direct descending from the adult stem cell [46,47,48].

## 5. The Challenge of Artificial Intelligence in Precision Medicine

The original objective of this “Commentary” was to point out several weaknesses of current approaches to understand the mechanisms of pathogenesis of human diseases via either methods of Artificial Intelligence (AI) for Precision Medicine or Hypothesis -driven mechanistic approaches. Using our current understanding of human carcinogenesis as an example. I have noted that several concepts and experiment findings have been seriously ignored in these recent attempts. Several old hypotheses, such as the “Stem cell Hypothesis”, role of cell–cell communication; threshold exposures to epigenetic acting chemicals, and biological characteristics of adult organ-stem cells are among those that need to be integrated into either algorithms of the AI approach (improving the Alan Turing model), or a more realistic concept of cancer as a whole animal phenomenon.

Starting from the beginning, the emergence of the family of the gap junction genes (connexins) [72]), among other related and supporting genes, allowed for the creation of a “society of cells” within a multicellular metazoan. The ability to regulate this family of genes within the other two more primitive cell signaling mechanisms allowed for homeostatic control of cell proliferation, differentiation, apoptosis, gene regulation, and senescence of cells in a 3-Dimensional setting. It must be remembered that in a multicellular metazoan, every organ consists of multiple cell types that must communicate by some signaling system within and between cell types. The existence of inherited mutations of various connexin genes has been associated with specific human diseases syndromes [73], as well as with knock out mice models [74], together with the mounting evidence linking specific epigenetic chemicals with all kinds of human diseases, from birth defects, cardiovascular diseases, immune disorders, reproductive- and neurological defects [75], has to be acknowledged at a critical cellular effect in predicting human diseases. Therefore, there must be mechanisms expressing specific connexin genes per unique cell types (each connexin protein is regulated at the transcription, translation and posttranslational level differently). This makes, especially with our knowledge at this time, our task to integrate all these complex interacting factors extremely difficult, if not impossible.

For the bio-informaticists and computer programmers, the simple task to integrate gap junctional intercellular communication into refining Alan Turnings algorithm will not be easy. This is because this system of communication, which makes possible homeostatic control in a whole organism is extremely dynamic [76]. It includes species, individual genetic, development stage, gender differences, as well the concepts of thresholds; length of time of exposures to epigenetic agents; absence of antioxidants; additivity, antagonistic and synergistic interactions in vivo. This integrated extra-, intra- and gap junctional intercellular communication system must be accounted for in any analysis for risk predictions in Precision Medicine. More “Real Intelligence” must be integrated into “Artificial Intelligence” for better risk predictions in Precision Medicine [77].

## References

[1] Gamma A. (2013). The role of genetic information in personalized medicine. Perspect. Biol. Med..

[2] Dubos R. (1969). Lasting biological effects of early influences. Perspect. Biol. Med..

[3] Collins F.S., Varmus H. (2015). A new initiative on precision medicine. N. Engl. J. Med..

[4] Turing A.M. (1952). The chemical basis of morphogenesis. Philos. Trans. R. Soc. Lond. Ser. B Biol. Sci..

[5] Loewenstein W.R., Kanno Y. (1966). Intercellular communication and the control of tissue growth: Lack of communication between cancer cells. Nature.

[6] Revel J.P., Yee A.G., Hudspeth A.J. (1971). Gap junctions between electrotonically coupled cells in tissue culture and in brown fat. Proc. Natl. Acad. Sci. USA.

[7] Klein C., Seelig F. (1995). Turing structures in a system with regulated gap-junctions. Biosystems.

[8] Deng C., Ji X., Rainey C., Zhang J., Lu W. (2020). Integrating machine learning with human knowledge. Iscience.

[9] Luo W., Phung Q.-D., Tran T., Gupta S., Rana S., Karmakar C., Shilton A., Yearwood J.L., Dimitrova N., Ho T.B. (2016). Guidelines for developing and reporting machine learning predictive models in biomedical research: A multidisciplinary view. J. Med. Internet Res..

[10] Furman S.A., Stern A.M., Uttam S., Taylor D.L., Pullara F., Chennubhotla S.C. (2021). In situ functional cell phenotyping reveals microdomain networks in colorectal cancer recurrence. Cell Rep. Methods.

[11] Trosko J.E. (2016). Evolution of microbial quorum sensing to human global quorum sensing: An insight into how gap junctional intercellular communication might be linked to the global metabolic disease crisis. Biology.

[12] Markert C.L. (1983). Genetic control of cell interactions in chimeras. Dev. Genet..

[13] Scadden D.T. (2006). The stem-cell niche as an entity of action. Nature.

[14] Mohyeldin A., Garzon-Muvdi T., Quiñones-Hinojosa A. (2010). Oxygen in stem cell biology: A critical component of the stem cell niche. PeerJ.

[15] Guo Y., Einhorn L., Kelley M., Hirota K., Yodoi J., Reinbold R., Scholer H., Ramsey H., Hromas R. (2004). Redox regulation of the embryonic stem cell transcription factor oct-4 by thioredoxin. Stem Cells.

[16] Mathias R.T., White T., Gong X. (2009). Lens gap junctions in growth, differentiation, and homeostasis. Physiol. Rev..

[17] Loewenstein W.R. (1981). Junctional intercellular communication: The cell to cell membrane channel. Physiol. Rev..

[18] Thomson J.A., Itskovitz-Eldor J., Shapiro S.S., Waknitz M.A., Swiergiel J.J., Marshall V.S., Jones J.M. (1998). Embryonic stem cell 500 lines derived from human blastocyst. Science.

[19] Shamblott M.J., Axelman J., Wang S., Bugg E.M., Littlefield J.W., Donovan P.J., Blumenthal P.D., Huggins G.R., Gearhart J.D. (1998). Derivation of pluripotent stem cells from cultured human primordial Germ cells. Proc. Natl. Acad. Sci. USA.

[20] Takahashi K., Yamanaka S. (2006). Induction of pluripotent stem cells from mouse embryonic and adult Fibroblast cultures by defined factors. Cell.

[21] Trosko J.E., Chang C.C., Wilson M.R., Upham B., Hayashi T., Wade M. (2000). Gap junctions and the regulation of cellular functions of stem cells during development and differentiation. Methods.

[22] Cantz T., Key G., Bleidiβel M., Gentile L., Han D.W., Brenne A., Scholer H.R. (2008). Absence of OCT4 expression in somatic tumor cell lines. Stem Cells.

[23] Berg J.S., Goodell M.A. (2007). An argument against a role for Oct4 in somatic stem cells. Cell Stem Cell.

[24] Lengner C., Camargo F.D., Hochedlinger K., Welstead G.G., Zaidi S., Gokhale S., Schöler H., Tomilin A., Jaenisch R. (2007). Oct4 expression is not required in mouse somatic stem cell self-renewal. Cell Stem Cell.

[25] Tai M.H., Chang C.C., Olson L.K., Trosko J.E. (2005). Oct4 expression in adult human stem cells: Evidence in support of the stem cell theory of carcinogenesis. Carcinogenesis.

[26] Weinstein I.B. (1987). Growth factors, oncogenes and multistage carcinogenesis. J. Cell Biochem..

[27] Pitot H.C., Dragan Y.P. (1991). Facts and theories concerning the mechanisms of carcinogenesis. FASEB J..

[28] Pitot H.C. (1989). Progression: The terminal stage in carcinogenesis. Jpn. J. Cancer Res..

[29] Hanahan D., Weinberg R.A. (2000). The hallmarks of cancer. Cell.

[30] Hanahan D., Weinberg R.A. (2011). Hallmarks of cancer: The next generation. Cell.

[31] Cleaver J.E., Trosko J.E. (1970). Absence of excision of ultraviolet-induced cyclobutane dimers in Xeroderma pigmentosum. Photochem. Photobiol..

[32] Maher V.M., McCormick J.J., Yuhas J.M., Tennant R.W., Regan J.D. (1976). Effect of DNA repair on the cytotoxicity and mutagenicity of UV irradiation and of chemical carcinogens in normal and xeroderma pigmentosum cells. Biology of Radiation Carcinogenesis.

[33] Glover T.W., Chang C.C., Trosko J.E., Li S.S. (1979). Ultraviolet light induction of diphtheria toxin resistant mutations in normal and xeroderma pigmentosum human fibroblasts. Proc. Natl. Acad. Sci. USA.

[34] Cleaver J.E. (1978). Xeroderma pigmentosum: Genetic and environmental influences in skin carcinogenesis. Int. J. Dermatol..

[35] Brash D.E., Rudolph J.A., Simon J.A., Lin A., McKenna G.J., Baden H.P., Halperin A.J., Ponten J. (1991). A role for sunlight in skin cancer: UV-induced p53 mutations in squamous cell carcinomas. Proc. Natl. Acad. Sci. USA.

[36] Warren S.T., Schultz R.A., Chang C.C., Wade M.H., Trosko J.E. (1981). Elevated spontaneous mutation rate in bloom syndrome fibroblasts. Proc. Natl. Acad. Sci. USA.

[37] Trosko J.E., Chang C.C., Hart R., Hoerger F.D. (1989). Nongenotoxic mechanisms in carcinogenesis: Role of inhibited intercellular communication. Banbury Report 31: New Directions in the Qualitative and Quantitative Aspects of Carcinogen Risk Assessment.

[38] Leone A., Longo C., Trosko J.E. (2012). The chemopreventive role of dietary phytochemicals through gap junctional intercellular communication. Phytochem. Rev..

[39] Trosko J.E. (2017). Reflections on the use of 10 IARC carcinogenic characteristics for an objective approach to identifying and organizing results from certain mechanistic studies. Toxicol. Res. Appl..

[40] Trosko J.E. (1998). Cell-cell communication in carcinogenesis. Front. Biosci..

[41] Ruch R.J. (2020). Gap junctions and connexins in cancer formation, progression, and therapy. Cancers.

[42] Markert C.L. (1968). Neoplasia: A disease of cell differentiation. Cancer Res..

[43] Potter V.R. (1978). Phenotypic diversity in experimental hepatomas: The concept of partially blocked ontogeny. Br. J. Cancer.

[44] Till J.E. (1982). Stem cells in differentiation and neoplasia. J. Cell. Physiol..

[45] Sell S. (1993). Cellular origin of cancer: Differentiation of stem cell maturation arrest?. Environ. Health Perspect..

[46] Trosko J.E. (2009). Cancer stem cells and cancer non-stem cells: From adult stem cells or from re-programming of differentiated somatic cells. Vet. Pathol..

[47] Trosko J.E. (2008). Human adult stem cells as the target cells for the initiation of carcinogenesis and for the generation of “cancer stem cells”. Int. J. Stem Cells.

[48] Trosko J.E. (2014). Induction of iPS cells and of cancer stem cells: The stem cell or reprogramming hypothesis of Cancer?. Anatom. Record.

[49] Chang C.C., Tsai J.L., Kuo K.K., Wang K.H., Chiang C.H., Kao A.P. (2004). Expression of Oct-4, alpha fetoprotein and vimentin, and lack of gap-junctional intercellular communication (GJIC) as common phenotypes for human adult liver stem cells and hepatoma cells. Proc. Amer. Assoc. Cancer Res..

[50] Zargari S., Khameneh S.N., Rad A., Forghanifard M.M. (2020). MEIS1 promotes expression of stem cell markers in esophageal squamous cell carcinomas. BMC Cancer.

[51] Nathansen J., Lukiyanchuk V., Hein L., Stolte M.-I., Borgmann K., Löck S., Kurth I., Baumann M., Krause M., Linge A. (2021). Oct4 confers stemness and radio-resistance to head and neck squamous carcinoma by regulation the homologous recombination factor PSMC31P and RAD54L. Oncogene.

[52] Ayoub M.M., Al-Sheikh SA M., Abdel-Salam LO A.F., Abdel-Moneimal-Hariry E.S.M. (2018). Expression of Oct4 protein in astrocytic tumors: Histological and immunohistochemical study. J. Clin. Diagn. Res..

[53] Noel K., Ibraheem M.M., Ahmed B.S., Hameed A.F., Khamees N.H., Akkila S.S. (2020). Expression of Oct4 stem cell marker in benign prostatic hyperplasia and normal tissue around the prostatic carcinoma in a sample of Iraqi patients. Egypt. J. Histol..

[54] Usta C.S., Turan G., Bulbul C.B., Usta A., Adali E. (2020). Differential expression of Oct4, CD44, and E-cadherin in eutopic and ectopic endometrium in ovarian endometriomas and their correlations with clinic pathological variables. Reprod. Biol. Endocrinol..

[55] Tegginamani A.S., Shivakumar V.H., Kallarakkal T.G., Ismail S.M., Abraham M.T., Zamzuri A.T.B. (2020). Analysis of octomer-binding transcription factor-4 expression in oral leukoplakia. J. Oral Maxillofac. Path..

[56] Villodre E.S., Kipper F.C., Pereira M.B., Lenz G. (2016). Roles of OCT4 in tumorigenesis, cancer therapy resistance and prognosis. Cancer Treat. Rev..

[57] Tsai L.-L., Yu C.-C., Chang Y.-C., Yu C.-H., Chou M.-Y. (2011). Markedly increased Oct4 and Nanog expression correlates with cisplatin resistance in oral squamous cell carcinoma. J. Oral Pathol. Med..

[58] Qiao B., He B., Cai J., Yang W. (2014). The expression profile of Oct4 and Sox2 in the carcinogenesis of oral mucosa. Int. J. Clin. Exp. Pathol..

[59] Hattermann K., Flüh C., Engel D., Mehdorn H.M., Synowitz M., Mentlein R., Held-Feindt J. (2016). Stem cell markers in glioma progression and recurrence. Int. J. Oncol..

[60] Zhang Q., Han Z., Zhu Y., Chen J., Li W. (2020). The role and specific mechanism of OCT4 in cancer stem cells: A review. Int. J. Stem Cells.

[61] Shao M., Bi T., Ding W., Yu C., Jiang C., Yang H., Sun X., Yang M. (2018). OCT4 potentiates radio-resistance and migration activity of rectal cancer cells by improving epithelial-mesenchymal transition in a ZEB1 dependent manner. BioMed Res. Int..

[62] Mohiuddin I.S., Wei S.J., Kang M.H. (2020). Role of OCT4 in cancer stem-like cells and chemotherapy resistance. Mol. Basis Dis..

[63] Wu G., Wilson G., Zhou G., Hebbard L., George J., Qiao L. (2015). Oct4 is a reliable marker of liver tumor propagating cells in hepatocellular carcinoma. Discov. Med..

[64] Zhou Y., Chen X., Kang B., She S., Zhang X., Chen C., Li W., Chen W., Dan S., Pan X. (2018). Endogenous authentic OCT4A proteins directly regulate FOS/AP-1 transcription in somatic cancer cells. Cell Death Dis..

[65] Rasti A., Mehrazma M., Madjd Z., Abolhasani M., Zanjani L.S., Asgari M. (2018). Co-expression of cancer stem cell markers OCT4 and NANOG predicts poor prognosis in renal cell carcinomas. Sci. Rep..

[66] Webster J.D., Yuzbasiyan-Gurkan V., Trosko J.E., Chang C.-C., Kiupel M. (2007). Expression of the embryonic transcription factor Oct4 in canine neoplasms: A potential marker for stem cell subpopulations in neoplasia. Vet. Pathol..

[67] Shimoda M., Ota M., Okada Y. (2018). Isolation of cancer stem cells by side population method. Methods Mol. Biol..

[68] Yamanaka S. (2012). Induced pluripotent stem cells: Past, present, and future. Cell Stem Cell.

[69] Toma J.G., McKenzie I.A., Bagli D., Miller F.D. (2005). Isolation and characterization of multipotent skin-derived precursors from humanskin. Stem Cells.

[70] Hochedlinge K. (2010). Cell type of origin influences the molecular and functional properties of mouse induced pluripotent stem cells. Nat. Biotechnol..

[71] Kim K., Doi A., Wen B., Ng K., Zhao R., Cahan P., Kim J., Aryee M.J., Ji H., Ehrlich L.I.R. (2010). Epigenetic memory in induced pluripotent stem cells. Nature.

[72] Cruciani V., Mikalsen S.-O. (2005). The connexin gene family in mammals. Biol. Chem..

[73] White T.W., Paul D.L. (1999). Genetic diseases and gene knockouts reveal diverse connexin functions. Annu. Rev. Physiol..

[74] Lo C.W. (2000). Role of gap junctions in cardiac conduction and development: Insights from the connexin knockout mice. Circul. Res..

[75] Trosko J.E., Wexler P. (2014). Gap junctional intercellular communication. Encyclopedia of Toxicology.

[76] Upham B.L., Trosko J.E. (2009). Oxidative-dependent integration of signal transduction with intercellular gap junctional communication in the control of gene expression. Antioxid. Redox Signal..

[77] Trosko J.E. (2021). On the potential origin and characteristics of cancer stem cells. Carcinogenesis.

